# Crystal structure of the tetra­ethyl­ammonium salt of the non-steroidal anti-inflammatory drug nimesulide (polymorph II)

**DOI:** 10.1107/S2056989024001300

**Published:** 2024-02-20

**Authors:** Małgorzata Rybczyńska, Artur Sikorski

**Affiliations:** a Faculty of Chemistry, University of Gdańsk, W. Stwosza 63, 80-308 Gdańsk, Poland; Indian Institute of Science Education and Research Bhopal, India

**Keywords:** nimesulide, *N*-(4-nitro-2-phen­oxy­phen­yl)methane­sulfonamide, tetra­ethyl­ammonium salt, API, crystal structure, polymorphism

## Abstract

The crystal structure of the tetra­ethyl­ammonium salt of the non-steroidal anti-inflammatory drug nimesulide (polymorph II) (systematic name: tetra­ethyl­ammonium *N*-methane­sulfonyl-4-nitro-2-phen­oxy­anilinide), C_8_H_20_N^+^·C_13_H_11_N_2_O_5_S^−^, was determined using single-crystal X-ray diffraction. The title compound crystallizes in the monoclinic space group *P*2_1_/*c* with one tetra­ethyl­ammonium cation and one nimesulide anion in the asymmetric unit. In the crystal, the ions are linked by C—H⋯N and C—H⋯O hydrogen bonds and C—H⋯π inter­actions. There are differences in the geometry of both the nimesulide anion and the tetra­ethyl­ammonium cation in polymorphs I [Rybczyńska & Sikorski (2023 ▸). *Sci. Rep.* **13**, 17268] and II of the title compound.

## Abstract 2

The crystal structure of the tetra­ethyl­ammonium salt of the non-steroidal anti-inflammatory drug nimesulide (polymorph II), C_8_H_20_N^+^·C_13_H_11_N_2_O_5_S^−^, was determined using single-crystal X-ray diffraction. There are differences in the geometry of both the nimesulide anion and the tetra­ethyl­ammonium cation in polymorphs I and II of the title compound.

## 1. Chemical context

Nimesulide [systematic name: *N*-(4-nitro-2-phen­oxy­phen­yl)methane­sulfonamide] is an active pharmaceutical ingredient (API) categorized among non-steroidal anti-inflammatory drugs (NSAIDs). This is a drug that effectively manages acute pain and primary dysmenorrhea as a result of its anti­pyretic, analgesic, and anti-inflammatory properties (Kress *et al.*, 2016 ▸; Vane & Botting, 1998 ▸). Similar to other NSAIDs, its action involves inhibiting cyclo­oxygenase – an enzyme crucial in prostaglandin synthesis within cell membranes (Bennett & Villa, 2000 ▸). 
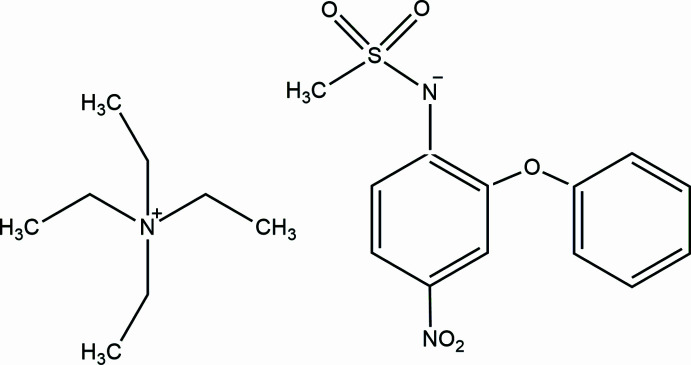


The crystal structure of nimesulide is known – it exists in the form of two polymorphs (Dupont *et al.*, 1995 ▸; Sanphui *et al.*, 2011 ▸; Banti *et al.*, 2016 ▸). However, only a few structures of multi-component crystals containing nimesulide have been described in the literature, such as co-crystals (Wang *et al.*, 2020 ▸) and metal complexes (Banti *et al.*, 2016 ▸), but only two, previously examined by us, structures of organic salts of nimesulide (Rybczyńska & Sikorski, 2023 ▸) are known. One of these salts is the tetra­ethyl­ammonium salt of nimesulide (polymorph I). We became inter­ested in it because the quaternary tetra­ethyl­ammonium cation has inter­esting bio­logical activities: it is a ganglionic blocker and inhibitor at nicotinic acetyl­choline (Kleinhaus & Prichard, 1977 ▸; Akk & Steinbach, 2003 ▸), and is a common organic structure-directing agent (OSDA) (Schmidt *et al.*, 2016 ▸).

In this research communication, as a continuation of our recent study on the tetra­alkyl­ammonium salts of nimesulide (Rybczyńska & Sikorski, 2023 ▸), we report on the crystal structure, conformational analysis of ions and analysis of inter­molecular inter­actions in the crystal of tetra­ethyl­ammonium salt of nimesulide (polymorph II).

## 2. Structural commentary

**Figure 1:**
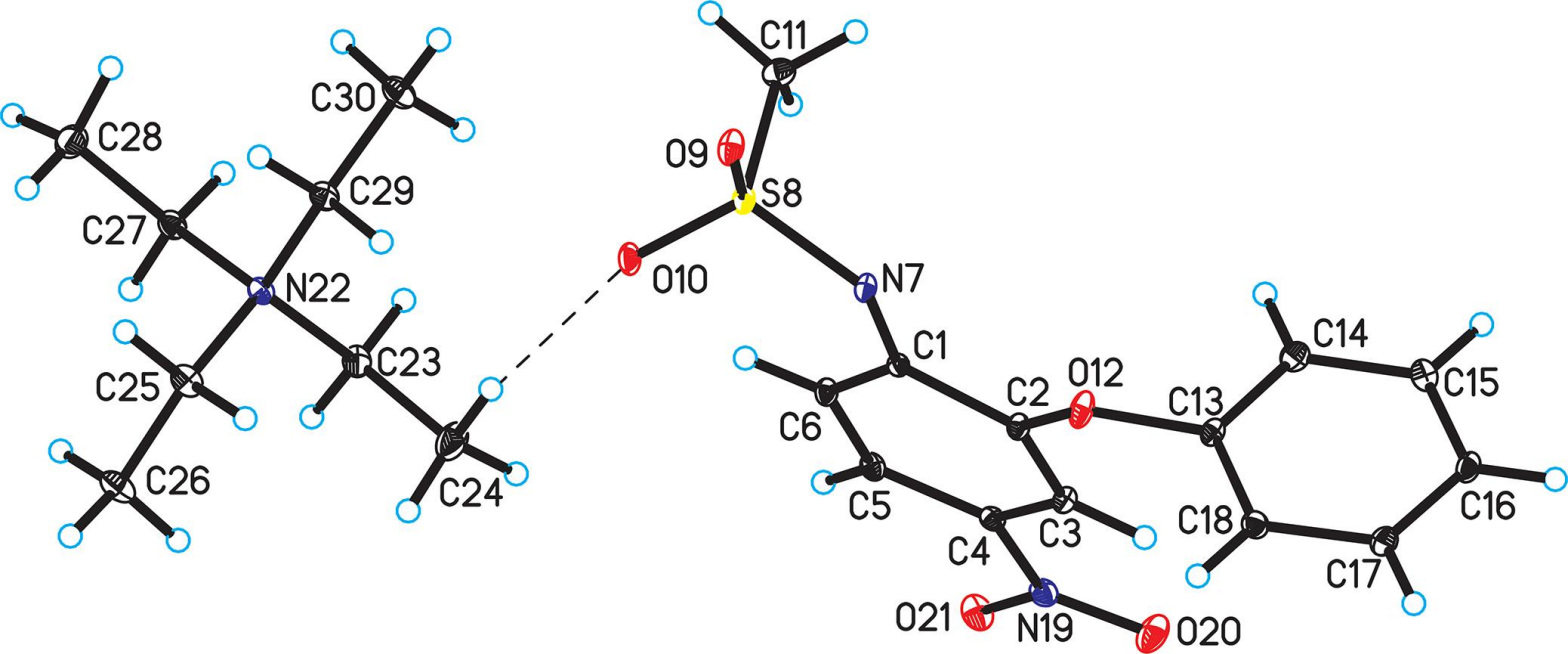
Crystal structure of title compound with the atom-labeling scheme (displacement ellipsoids are drawn at the 25% probability level; hydrogen bonds are represented by dashed lines).

The title compound crystallizes in the monoclinic *P*2_1_/*c* space group with one tetra­ethyl­ammonium cation and one nimesulide anion in the asymmetric unit (Table 1 ▸, Fig. 1 ▸). For comparison, polymorph I crystallizes in the monoclinic *P*2_1_/*n* space group with one ion pair in the asymmetric unit.

**Figure 2:**
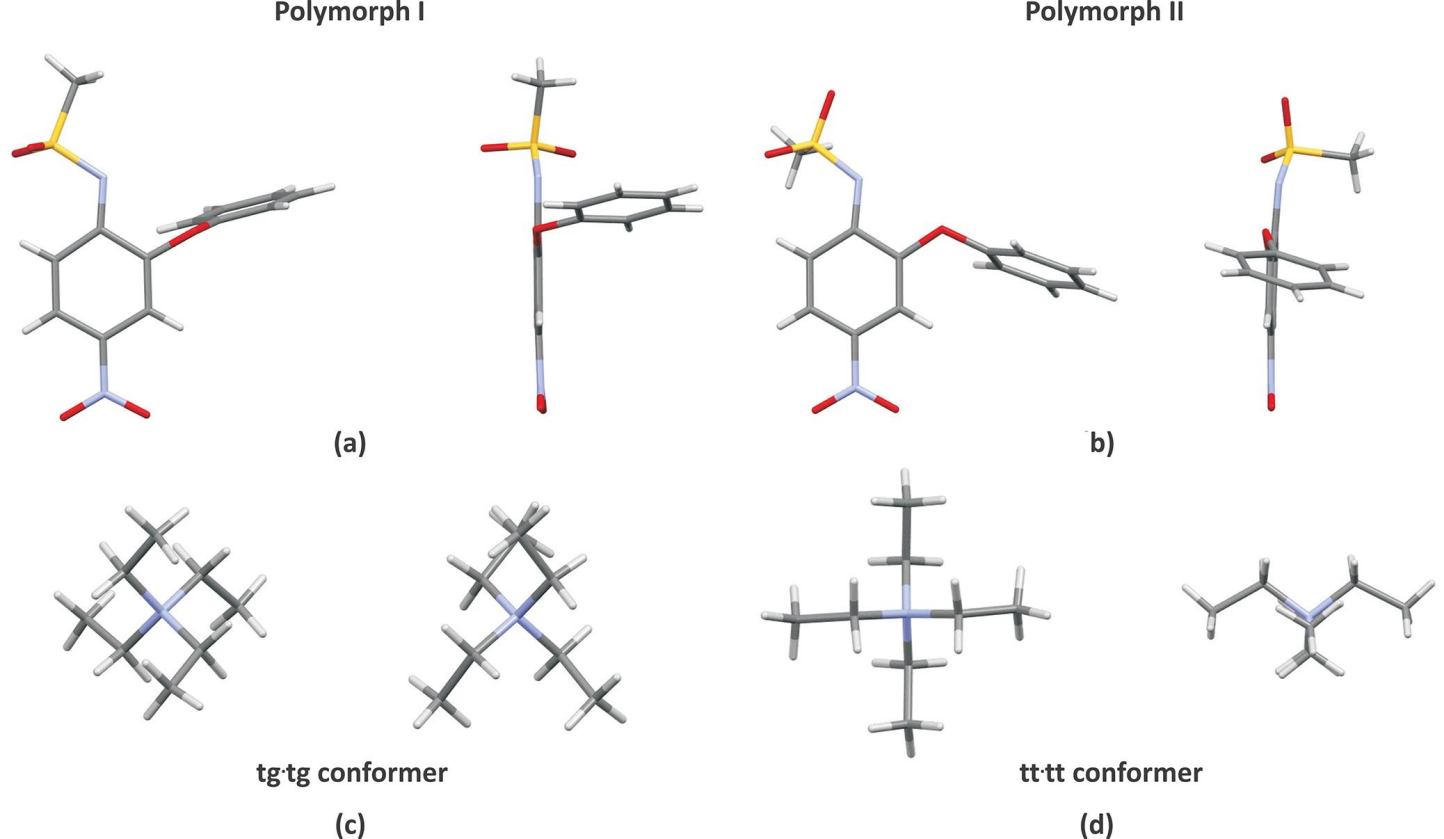
Comparison of the geometries of the nimesulide anion (*a*) and (*b*) and the tetra­ethyl­ammonium cation (*c*) and (*d*) in the crystals of the two polymorphs of the tetra­ethyl­ammonium salt of nimesulide.

In the crystal structure of the title compound, nimesulide occurs in an ionized form, which is confirmed by the C1—N7 [*d*(C—N) = 1.365 (4) Å] and N7—S8 [*d*(N—S) = 1.584 (2) Å] bond lengths and the value of the C1—N7—S8 angle [∠(C—N—S) = 122.7 (2)°] in the sulfonamide group. Similar *d*(N—S) values are also observed in the crystal structure of polymorph I [1.589 (2) Å], but the *d*(C—N) distance is slightly shorter and the ∠(C—N—S) angle is smaller for polymorph I [1.345 (3) Å and 119.2 (2)°, respectively]. There are also differences in the arrangement of the methyl group from the sulfonamide moiety and the phen­oxy group within the nimesulide anion (Fig. 2 ▸). In the crystal of polymorph I, the methyl group lies almost in the plane of the phenyl ring of the nimesulide anion [with torsion angle ∠(C1—N7—S8—C11) = −174.7 (2)°], while in the crystal of polymorph II it is almost perpendicular [torsion angle ∠(C1—N7—S8—C11) = −74.0 (3)°]. In turn, in the crystal of polymorph I, the phen­oxy group is tilted and twisted relative to the benzene ring of nimesulide, with a torsion angle of ∠ (C3—C2—O12—C13) = 88.5 (2)° and an inter­planar angle of 84.8 (2)°, while in the crystal of polymorph II the values of these angles are 20.9(4 and 78.3 (2)°, respectively.

Differences in the geometry of the tetra­ethyl­ammonium cation in the crystals of the two polymorphs of the title compound are also observed (Fig. 2 ▸). In the case of polymorph I, the cation adopts the geometry of a *tg*·*tg* conformer, while in the crystal of polymorph II it exists in a *tt*·*tt* conformer (Ikuno *et al.*, 2015 ▸; Schmidt *et al.*, 2016 ▸; Takekiyo & Yoshimura, 2006 ▸). Both conformers of the tetra­ethyl­ammonium cation are also observed in other tetra­ethyl­ammonium salts (*e.g.* de Arriba *et al.*, 2011 ▸; Evans *et al.*, 1990 ▸; Warnke *et al.*, 2010 ▸; Lutz *et al.*, 2014 ▸; Brahim *et al.*, 2018 ▸). It is inter­esting that the distribution of conformers of the tetra­ethyl­ammonium cation in tetra­ethyl­ammonium hydroxide solution is temperature dependent (the *tt*·*tt* conformer dominates at lower temperatures), and higher concentrations lead to a greater proportion of the *tg*·*tg* conformer (Ikuno *et al.*, 2015 ▸; Schmidt *et al.*, 2016 ▸; Takekiyo & Yoshimura, 2006 ▸). This may explain why only a few single crystals of polymorph II were obtained as a result of the synthesis of the title compound carried out under specific conditions (see: *Synthesis and crystallization* section).

The changes in the conformation of both the nimesulide anion and the tetra­ethyl­ammonium cation results in an increase in the volume of the unit cell from 2300.6 (2) Å^3^ (polymorph I) to 2330.0 (4) Å^3^ (polymorph II). Moreover, the crystal density decreases (1.292 and 1.272 g cm^−3^ for polymorph I and II, respectively), as well as the Kitaigorodskii packing index (with the percentage of filled space equal to 66.7 and 66.0% for polymorphs I and II, respectively). This indicates a more favorable mol­ecular packing in the crystal of polymorph I.

## 3. Supra­molecular features

**Figure 3:**
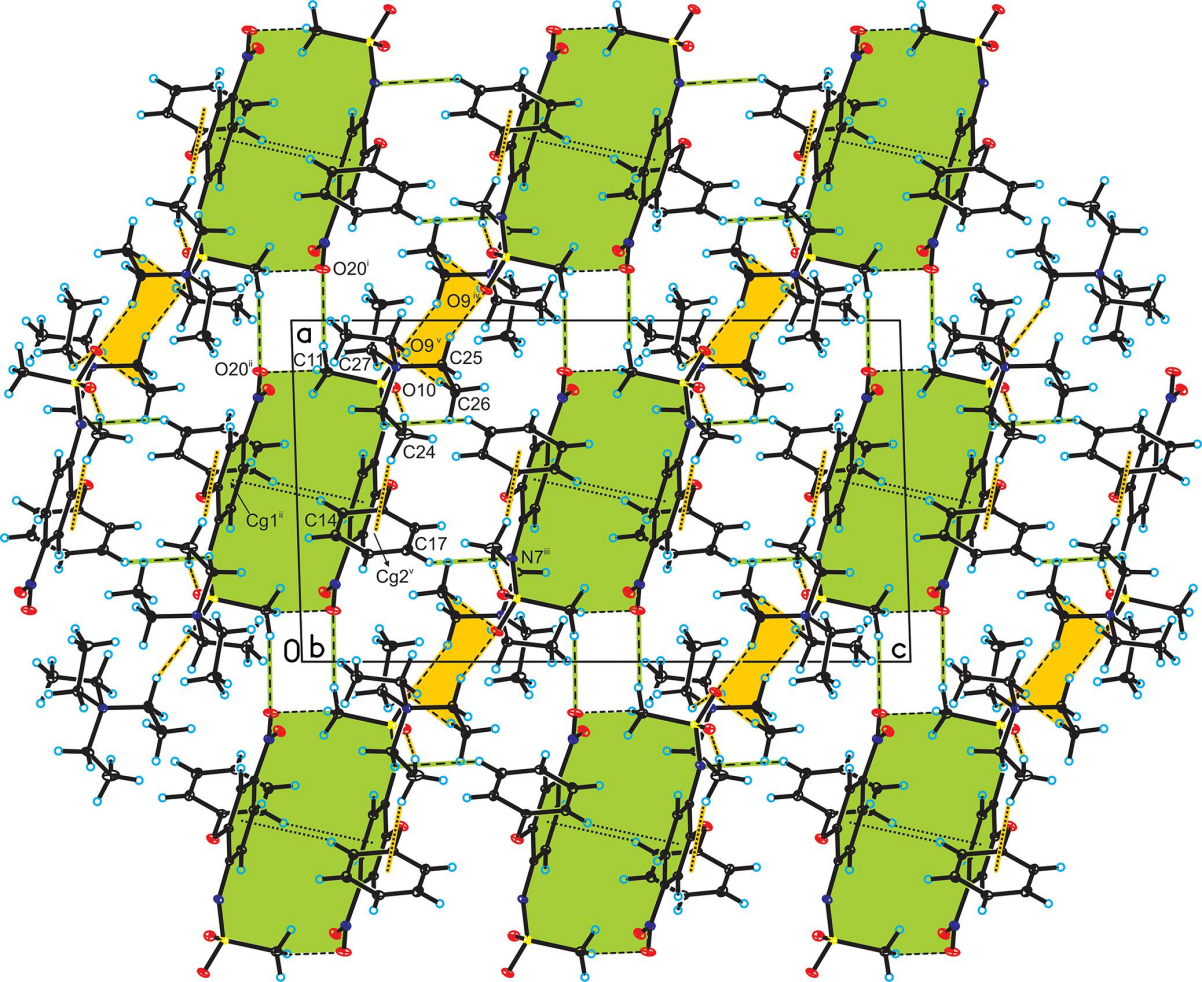
Crystal packing of the title compound viewed along the *b* axis (inter­actions between nimesulide anions are highlighted in green, whereas inter­actions between the nimesulide anion and tetra­ethyl­ammonium cation are highlighted in orange).

In the crystal of the title compound, neighboring nimesulide anions are linked by C14—H14*A*⋯π inter­actions [*d*(H⋯*Cg*) = 3.07 Å; Fig. 3 ▸, Table 1 ▸], forming a homodimer. Adjacent homodimers are linked through C_phen­oxy_—H⋯N^−^ and C_meth­yl_—H⋯O_nitro_ hydrogen bonds, building porous organic frameworks along the *b*-axis (Fig. 3 ▸, Table 1 ▸). The tetra­ethyl­ammonium cations are located in the voids of these networks and linked with the nimesulide anions *via* C_meth­yl_—H⋯O_sulfo_ hydrogen bonds and C24—H24*A*⋯π_phen­oxy_ inter­actions [*d*(H⋯*Cg*) = 2.88 Å; Fig. 3 ▸, Table 1 ▸].

## 4. Database survey

In the Cambridge Structural Database (CSD version 5.43, update of 03/2023; Groom *et al.*, 2016 ▸) there are only 13 structures involving a nimesulide mol­ecule or ion, *viz*., the crystal structures of two polymorphs of nimesulide [refcodes WINWUL (Dupont *et al.*, 1995 ▸), WINWUL01, WINWUL02 (Sanphui *et al.*, 2011 ▸), and WINWUL03 (Banti *et al.*, 2016 ▸)], five structures of nimesulide–silver complexes (refcodes EXEZUE, EXIBAQ, EXIBAU, EXIBIY, EXIBOE; Banti *et al.*, 2016 ▸), the crystal structures of tetra­methyl­ammonium and tetra­ethyl­ammonium salts of nimesulide (polymorph I; CCDC 2281374 and CCDC 2281375; Rybczyńska & Sikorski, 2023 ▸), and four structures of co-crystals of nimesulide with pyridine derivatives (refcodes LAKLOC, LAKLUI, LAKMAP, and LAKMET; Wang *et al.*, 2021 ▸). In the CSD, there are also 5062 structures of tetra­ethyl­ammonium salts: 728 of them are structures of organic compounds involving the tetra­ethyl­ammonium cation, including three structures of sulfonamide salts (refcodes RALGOC, RALGUI, and RALHAP; de Arriba *et al.*, 2011 ▸).

## 5. Synthesis and crystallization

All chemicals were purchased from Sigma-Aldrich and used without any further purification. Nimesulide (0.05 g, 0.162 mmol) was dissolved in 0.12 ml of tetra­ethyl­ammonium hydroxide (20 wt.% in H_2_O, *d* = 1.01 g cm^−3^ in 293 K, 0.162 mmol) and 5 cm^3^ of ethanol. The solution was mixed and heated until boiling. The solution was allowed to evaporate in place without sunlight for a few days, giving yellow crystals of polymorph I and a small amount of yellow crystals of polymorph II (m.p. = 388 K). The mixture of polymorphs was separated by mechanical means.

## 6. Refinement

Crystal data, data collection and structure refinement details are summarized in Table 2 ▸. All H atoms were placed geom­etrically and refined using a riding model with C—H = 0.93–0.97 Å and *U*_iso_(H) = 1.2*U*_eq_(C) [C—H = 0.96 Å and *U*_iso_(H) = 1.5*U*_eq_(C) for the methyl groups]. The most disagreeable reflections (621) and (589) with an error/s.u. of more than 10 were omitted using the OMIT instruction in *SHELXL* (Sheldrick, 2015*b* ▸).

## Supplementary Material

Crystal structure: contains datablock(s) I. DOI: 10.1107/S2056989024001300/dx2059sup1.cif

Structure factors: contains datablock(s) I. DOI: 10.1107/S2056989024001300/dx2059Isup2.hkl

Supporting information file. DOI: 10.1107/S2056989024001300/dx2059Isup3.mol

Supporting information file. DOI: 10.1107/S2056989024001300/dx2059Isup4.cml

CCDC reference: 2332021

Additional supporting information:  crystallographic information; 3D view; checkCIF report

## Figures and Tables

**Table 1:** Hydrogen-bond geometry (Å, °) *Cg*1 and *Cg*2 are the centroids of the C1–C6 and C13–C18 rings, respectively.

*D*—H⋯*A*	*D*—H	H⋯*A*	*D*⋯*A*	*D*—H⋯*A*
C11—H11*A*⋯O20^i^	0.96	2.65	3.360 (4)	131
C11—H11*C*⋯O20^ii^	0.96	2.66	3.555 (4)	156
C17—H17*A*⋯N7^iii^	0.93	2.70	3.478 (4)	142
C24—H24*C*⋯O10	0.96	2.47	3.415 (4)	168
C25—H25*A*⋯O9^iv^	0.97	2.47	3.155 (5)	128
C26—H26*C*⋯O9^v^	0.96	2.58	3.541 (4)	175
C27—H27*B*⋯O9^v^	0.97	2.52	3.267 (4)	134
C14—H14*A*⋯*Cg*1^ii^	0.93	3.07	3.951 (5)	158
C24—H24*A*⋯*Cg*2^v^	0.96	2.88	3.608 (5)	134

Symmetry codes: (i) 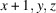 ; (ii) 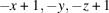 ; (iii) 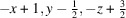 ; (iv) 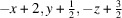 ; (v) 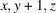 .

**Table 2:** Experimental details

Crystal data	
Chemical formula	C_8_H_20_N^+^·C_13_H_11_N_2_O_5_S^−^
*M*_r_	437.55
Crystal system, space group	Monoclinic, *P*2_1_/*c*
Temperature (K)	291
*a*, *b*, *c* (Å)	11.0276 (10), 10.7661 (8), 19.635 (2)
β (°)	91.792 (9)
*V* (Å^3^)	2330.0 (4)
*Z*	4
Radiation type	Mo *K*α
μ (mm^−1^)	0.17
Crystal size (mm)	0.42 × 0.20 × 0.09

Data collection	
Diffractometer	Oxford Diffraction Ruby CCD
Absorption correction	Multi-scan (*CrysAlis RED*; Oxford Diffraction, 2008 ▸).
*T*_min_, *T*_max_	0.966, 0.998
No. of measured, independent and observed [*I* > 2σ(*I*)] reflections	15531, 4094, 2549
*R*_int_	0.073
(sin θ/λ)_max_ (Å^−1^)	0.595

Refinement	
*R*[*F* ^2^ > 2σ(*F* ^2^)], *wR*(*F* ^2^), *S*	0.066, 0.128, 1.10
No. of reflections	4094
No. of parameters	276
H-atom treatment	H-atom parameters constrained
Δρ_max_, Δρ_min_ (e Å^−3^)	0.16, −0.23

Computer programs: *CrysAlis CCD* and *CrysAlis RED* (Oxford Diffraction, 2008 ▸), SHELXT (Sheldrick, 2015*a* ▸), *SHELXL2017/1* (Sheldrick, 2015*b* ▸), *ORTEP-3 for Windows* (Farrugia, 2012 ▸), and *publCIF* (Westrip, 2010 ▸).

## supplementary crystallographic information

### Crystal data

**Table:**

C_8_H_20_N^+^·C_13_H_11_N_2_O_5_S^−^	*D*_x_ = 1.247 Mg m^−^^3^
*M**_r_* = 437.55	Melting point: 388 K
Monoclinic, *P*2_1_/*c*	Mo *K*α radiation, λ = 0.71073 Å
*a* = 11.0276 (10) Å	Cell parameters from 15531 reflections
*b* = 10.7661 (8) Å	θ = 3.3–25.0°
*c* = 19.635 (2) Å	µ = 0.17 mm^−^^1^
β = 91.792 (9)°	*T* = 291 K
*V* = 2330.0 (4) Å^3^	Plate, yellow
*Z* = 4	0.42 × 0.20 × 0.09 mm
*F*(000) = 936	

### Data collection

**Table:**

Oxford Diffraction Ruby CCD diffractometer	4094 independent reflections
Radiation source: fine-focus sealed tube	2549 reflections with *I* > 2σ(*I*)
Graphite monochromator	*R*_int_ = 0.073
Detector resolution: 10.4002 pixels mm^-1^	θ_max_ = 25.0°, θ_min_ = 3.3°
ω scans	*h* = −13→12
Absorption correction: multi-scan (CrysAlis RED; Oxford Diffraction, 2008).	*k* = −12→11
*T*_min_ = 0.966, *T*_max_ = 0.998	*l* = −23→23
15531 measured reflections	

### Refinement

**Table:**

Refinement on *F*^2^	Primary atom site location: difference Fourier map
Least-squares matrix: full	Hydrogen site location: inferred from neighbouring sites
*R*[*F*^2^ > 2σ(*F*^2^)] = 0.066	H-atom parameters constrained
*wR*(*F*^2^) = 0.128	*w* = 1/[σ^2^(*F*_o_^2^) + (0.0316*P*)^2^ + 0.0136*P*] where *P* = (*F*_o_^2^ + 2*F*_c_^2^)/3
*S* = 1.10	(Δ/σ)_max_ = 0.001
4094 reflections	Δρ_max_ = 0.16 e Å^−^^3^
276 parameters	Δρ_min_ = −0.23 e Å^−^^3^
0 restraints	

### Special details

**Table:**

Geometry. All esds (except the esd in the dihedral angle between two l.s. planes) are estimated using the full covariance matrix. The cell esds are taken into account individually in the estimation of esds in distances, angles and torsion angles; correlations between esds in cell parameters are only used when they are defined by crystal symmetry. An approximate (isotropic) treatment of cell esds is used for estimating esds involving l.s. planes.

### Fractional atomic coordinates and isotropic or equivalent isotropic displacement parameters (Å^2^)

**Table:**

	*x*	*y*	*z*	*U*_iso_*/*U*_eq_	
N22	0.8646 (2)	0.6786 (2)	0.66998 (12)	0.0466 (7)	
C23	0.7364 (3)	0.6589 (3)	0.6432 (2)	0.0679 (10)	
H23A	0.738581	0.638164	0.595204	0.082*	
H23B	0.692237	0.736376	0.647115	0.082*	
C24	0.6669 (4)	0.5566 (3)	0.6801 (3)	0.1112 (17)	
H24A	0.590665	0.541912	0.656474	0.167*	
H24B	0.652626	0.582332	0.725925	0.167*	
H24C	0.713924	0.481527	0.680791	0.167*	
C25	0.8677 (4)	0.7082 (3)	0.74529 (16)	0.0678 (10)	
H25A	0.951411	0.722287	0.759958	0.081*	
H25B	0.839097	0.636099	0.769643	0.081*	
C26	0.7934 (4)	0.8196 (3)	0.76588 (18)	0.0801 (12)	
H26A	0.804123	0.833377	0.814002	0.120*	
H26B	0.709188	0.804419	0.755044	0.120*	
H26C	0.819681	0.891737	0.741652	0.120*	
C27	0.9144 (3)	0.7874 (3)	0.63015 (17)	0.0609 (10)	
H27A	0.905286	0.768693	0.581930	0.073*	
H27B	0.865104	0.859849	0.638968	0.073*	
C28	1.0453 (4)	0.8203 (3)	0.6455 (2)	0.0968 (15)	
H28A	1.064308	0.897193	0.623559	0.145*	
H28B	1.096702	0.755682	0.628966	0.145*	
H28C	1.058343	0.828854	0.693876	0.145*	
C29	0.9416 (3)	0.5635 (3)	0.66162 (18)	0.0599 (9)	
H29A	1.021525	0.579523	0.681782	0.072*	
H29B	0.905901	0.496372	0.687120	0.072*	
C30	0.9563 (4)	0.5207 (3)	0.58923 (19)	0.0810 (12)	
H30A	1.002975	0.445354	0.589154	0.122*	
H30B	0.997549	0.583607	0.564185	0.122*	
H30C	0.877894	0.505702	0.568264	0.122*	
C1	0.5837 (3)	0.1058 (3)	0.62935 (14)	0.0395 (7)	
C2	0.4877 (3)	0.0172 (3)	0.62920 (15)	0.0435 (8)	
C3	0.3730 (3)	0.0461 (3)	0.60651 (15)	0.0487 (8)	
H3A	0.312492	−0.014135	0.605923	0.058*	
C4	0.3469 (3)	0.1659 (3)	0.58428 (15)	0.0443 (8)	
C5	0.4352 (3)	0.2565 (3)	0.58697 (16)	0.0508 (9)	
H5A	0.416335	0.337348	0.573719	0.061*	
C6	0.5507 (3)	0.2275 (3)	0.60917 (15)	0.0482 (8)	
H6A	0.609174	0.289699	0.611006	0.058*	
N7	0.6961 (2)	0.0639 (2)	0.64924 (12)	0.0463 (7)	
S8	0.81473 (7)	0.14575 (7)	0.64359 (4)	0.0480 (2)	
O9	0.9111 (2)	0.07799 (19)	0.67799 (12)	0.0651 (7)	
O10	0.8031 (2)	0.27336 (18)	0.66560 (12)	0.0650 (7)	
C11	0.8486 (3)	0.1503 (3)	0.55687 (17)	0.0700 (10)	
H11A	0.923935	0.193215	0.551381	0.105*	
H11B	0.784964	0.192962	0.531955	0.105*	
H11C	0.855381	0.067033	0.539846	0.105*	
O12	0.5189 (2)	−0.09796 (19)	0.65561 (12)	0.0652 (7)	
C13	0.4449 (3)	−0.2003 (3)	0.63959 (18)	0.0470 (8)	
C14	0.4353 (3)	−0.2436 (3)	0.57443 (18)	0.0627 (10)	
H14A	0.471834	−0.201598	0.539124	0.075*	
C15	0.3699 (3)	−0.3516 (3)	0.56192 (18)	0.0666 (10)	
H15A	0.362559	−0.382462	0.517737	0.080*	
C16	0.3162 (3)	−0.4132 (3)	0.6137 (2)	0.0615 (10)	
H16A	0.272162	−0.485449	0.604827	0.074*	
C17	0.3275 (3)	−0.3681 (3)	0.6785 (2)	0.0681 (11)	
H17A	0.291625	−0.410340	0.713952	0.082*	
C18	0.3917 (3)	−0.2604 (3)	0.69191 (17)	0.0583 (9)	
H18A	0.398465	−0.229272	0.736042	0.070*	
N19	0.2270 (3)	0.1945 (3)	0.55838 (14)	0.0586 (8)	
O20	0.1507 (2)	0.1114 (3)	0.55356 (16)	0.0935 (9)	
O21	0.2031 (2)	0.3022 (2)	0.54080 (13)	0.0770 (8)	

### Atomic displacement parameters (Å^2^)

**Table:**

	*U*^11^	*U*^22^	*U*^33^	*U*^12^	*U*^13^	*U*^23^
N22	0.0522 (17)	0.0362 (15)	0.0510 (16)	0.0145 (12)	−0.0039 (14)	0.0031 (12)
C23	0.050 (2)	0.054 (2)	0.098 (3)	0.0153 (18)	−0.014 (2)	−0.007 (2)
C24	0.073 (3)	0.056 (3)	0.205 (5)	−0.003 (2)	0.014 (3)	0.001 (3)
C25	0.087 (3)	0.059 (2)	0.056 (2)	0.018 (2)	−0.007 (2)	0.0037 (18)
C26	0.119 (4)	0.061 (2)	0.061 (2)	0.024 (2)	0.015 (2)	−0.0029 (19)
C27	0.074 (3)	0.045 (2)	0.064 (2)	0.0070 (18)	0.007 (2)	−0.0001 (17)
C28	0.072 (3)	0.068 (3)	0.151 (4)	−0.002 (2)	0.021 (3)	−0.006 (3)
C29	0.058 (2)	0.0418 (19)	0.080 (2)	0.0192 (17)	0.000 (2)	−0.0001 (17)
C30	0.097 (3)	0.055 (2)	0.091 (3)	0.024 (2)	0.009 (3)	−0.011 (2)
C1	0.040 (2)	0.0374 (17)	0.0410 (17)	0.0008 (15)	−0.0005 (15)	−0.0015 (14)
C2	0.044 (2)	0.0340 (18)	0.0525 (19)	0.0003 (15)	−0.0052 (16)	0.0032 (15)
C3	0.042 (2)	0.0476 (19)	0.056 (2)	−0.0055 (16)	−0.0017 (17)	0.0028 (16)
C4	0.040 (2)	0.045 (2)	0.0471 (18)	0.0125 (16)	−0.0036 (15)	−0.0040 (15)
C5	0.057 (2)	0.0385 (19)	0.057 (2)	0.0086 (17)	−0.0053 (18)	−0.0015 (15)
C6	0.050 (2)	0.0336 (18)	0.061 (2)	−0.0008 (15)	−0.0035 (18)	0.0000 (15)
N7	0.0420 (16)	0.0359 (14)	0.0604 (16)	−0.0043 (12)	−0.0097 (13)	0.0023 (12)
S8	0.0430 (5)	0.0379 (5)	0.0625 (5)	−0.0019 (4)	−0.0088 (4)	−0.0006 (4)
O9	0.0511 (15)	0.0513 (14)	0.0908 (17)	−0.0002 (11)	−0.0303 (13)	0.0074 (12)
O10	0.0650 (16)	0.0354 (13)	0.0943 (18)	−0.0065 (11)	−0.0034 (14)	−0.0120 (12)
C11	0.057 (2)	0.083 (3)	0.070 (2)	0.004 (2)	0.003 (2)	0.005 (2)
O12	0.0570 (15)	0.0397 (13)	0.0973 (18)	−0.0101 (11)	−0.0246 (13)	0.0216 (12)
C13	0.041 (2)	0.0308 (17)	0.069 (2)	0.0017 (15)	−0.0028 (18)	0.0121 (17)
C14	0.069 (3)	0.060 (2)	0.059 (2)	−0.005 (2)	0.005 (2)	0.0146 (19)
C15	0.077 (3)	0.058 (2)	0.064 (2)	−0.002 (2)	−0.005 (2)	−0.008 (2)
C16	0.053 (2)	0.0384 (19)	0.093 (3)	−0.0068 (16)	0.000 (2)	0.004 (2)
C17	0.074 (3)	0.052 (2)	0.079 (3)	−0.014 (2)	0.020 (2)	0.006 (2)
C18	0.069 (2)	0.048 (2)	0.058 (2)	−0.0018 (18)	0.009 (2)	0.0001 (17)
N19	0.053 (2)	0.059 (2)	0.0638 (18)	0.0120 (17)	−0.0041 (16)	−0.0079 (16)
O20	0.0448 (17)	0.0792 (19)	0.155 (3)	−0.0002 (15)	−0.0180 (17)	0.0119 (18)
O21	0.0763 (19)	0.0619 (17)	0.0912 (18)	0.0275 (14)	−0.0240 (15)	−0.0052 (14)

### Geometric parameters (Å, º)

**Table:**

N22—C23	1.508 (4)	C2—C3	1.364 (4)
N22—C25	1.512 (4)	C2—O12	1.383 (3)
N22—C29	1.514 (3)	C3—C4	1.389 (4)
N22—C27	1.520 (4)	C3—H3A	0.9300
C23—C24	1.536 (5)	C4—C5	1.378 (4)
C23—H23A	0.9700	C4—N19	1.436 (4)
C23—H23B	0.9700	C5—C6	1.370 (4)
C24—H24A	0.9600	C5—H5A	0.9300
C24—H24B	0.9600	C6—H6A	0.9300
C24—H24C	0.9600	N7—S8	1.584 (2)
C25—C26	1.515 (4)	S8—O9	1.439 (2)
C25—H25A	0.9700	S8—O10	1.447 (2)
C25—H25B	0.9700	S8—C11	1.755 (3)
C26—H26A	0.9600	C11—H11A	0.9600
C26—H26B	0.9600	C11—H11B	0.9600
C26—H26C	0.9600	C11—H11C	0.9600
C27—C28	1.508 (5)	O12—C13	1.401 (3)
C27—H27A	0.9700	C13—C14	1.363 (4)
C27—H27B	0.9700	C13—C18	1.363 (4)
C28—H28A	0.9600	C14—C15	1.387 (5)
C28—H28B	0.9600	C14—H14A	0.9300
C28—H28C	0.9600	C15—C16	1.364 (5)
C29—C30	1.508 (4)	C15—H15A	0.9300
C29—H29A	0.9700	C16—C17	1.365 (5)
C29—H29B	0.9700	C16—H16A	0.9300
C30—H30A	0.9600	C17—C18	1.379 (4)
C30—H30B	0.9600	C17—H17A	0.9300
C30—H30C	0.9600	C18—H18A	0.9300
C1—N7	1.365 (4)	N19—O20	1.230 (3)
C1—C6	1.412 (4)	N19—O21	1.235 (3)
C1—C2	1.425 (4)		
			
C23—N22—C25	111.3 (3)	N7—C1—C6	127.6 (3)
C23—N22—C29	111.7 (2)	N7—C1—C2	116.6 (3)
C25—N22—C29	106.5 (2)	C6—C1—C2	115.8 (3)
C23—N22—C27	106.2 (2)	C3—C2—O12	123.0 (3)
C25—N22—C27	110.1 (2)	C3—C2—C1	122.0 (3)
C29—N22—C27	111.2 (2)	O12—C2—C1	115.0 (3)
N22—C23—C24	114.4 (3)	C2—C3—C4	119.7 (3)
N22—C23—H23A	108.7	C2—C3—H3A	120.2
C24—C23—H23A	108.7	C4—C3—H3A	120.2
N22—C23—H23B	108.7	C5—C4—C3	120.4 (3)
C24—C23—H23B	108.7	C5—C4—N19	120.2 (3)
H23A—C23—H23B	107.6	C3—C4—N19	119.4 (3)
C23—C24—H24A	109.5	C6—C5—C4	120.0 (3)
C23—C24—H24B	109.5	C6—C5—H5A	120.0
H24A—C24—H24B	109.5	C4—C5—H5A	120.0
C23—C24—H24C	109.5	C5—C6—C1	122.0 (3)
H24A—C24—H24C	109.5	C5—C6—H6A	119.0
H24B—C24—H24C	109.5	C1—C6—H6A	119.0
N22—C25—C26	115.6 (3)	C1—N7—S8	122.7 (2)
N22—C25—H25A	108.4	O9—S8—O10	114.35 (14)
C26—C25—H25A	108.4	O9—S8—N7	106.51 (13)
N22—C25—H25B	108.4	O10—S8—N7	115.20 (13)
C26—C25—H25B	108.4	O9—S8—C11	107.03 (16)
H25A—C25—H25B	107.4	O10—S8—C11	106.68 (16)
C25—C26—H26A	109.5	N7—S8—C11	106.53 (16)
C25—C26—H26B	109.5	S8—C11—H11A	109.5
H26A—C26—H26B	109.5	S8—C11—H11B	109.5
C25—C26—H26C	109.5	H11A—C11—H11B	109.5
H26A—C26—H26C	109.5	S8—C11—H11C	109.5
H26B—C26—H26C	109.5	H11A—C11—H11C	109.5
C28—C27—N22	115.9 (3)	H11B—C11—H11C	109.5
C28—C27—H27A	108.3	C2—O12—C13	119.0 (2)
N22—C27—H27A	108.3	C14—C13—C18	121.5 (3)
C28—C27—H27B	108.3	C14—C13—O12	120.5 (3)
N22—C27—H27B	108.3	C18—C13—O12	117.8 (3)
H27A—C27—H27B	107.4	C13—C14—C15	118.6 (3)
C27—C28—H28A	109.5	C13—C14—H14A	120.7
C27—C28—H28B	109.5	C15—C14—H14A	120.7
H28A—C28—H28B	109.5	C16—C15—C14	120.8 (3)
C27—C28—H28C	109.5	C16—C15—H15A	119.6
H28A—C28—H28C	109.5	C14—C15—H15A	119.6
H28B—C28—H28C	109.5	C15—C16—C17	119.5 (3)
C30—C29—N22	115.5 (3)	C15—C16—H16A	120.2
C30—C29—H29A	108.4	C17—C16—H16A	120.2
N22—C29—H29A	108.4	C16—C17—C18	120.6 (3)
C30—C29—H29B	108.4	C16—C17—H17A	119.7
N22—C29—H29B	108.4	C18—C17—H17A	119.7
H29A—C29—H29B	107.5	C13—C18—C17	119.1 (3)
C29—C30—H30A	109.5	C13—C18—H18A	120.5
C29—C30—H30B	109.5	C17—C18—H18A	120.5
H30A—C30—H30B	109.5	O20—N19—O21	121.5 (3)
C29—C30—H30C	109.5	O20—N19—C4	119.4 (3)
H30A—C30—H30C	109.5	O21—N19—C4	119.1 (3)
H30B—C30—H30C	109.5		
			
C25—N22—C23—C24	−57.0 (4)	N7—C1—C6—C5	176.7 (3)
C29—N22—C23—C24	61.9 (4)	C2—C1—C6—C5	−3.8 (4)
C27—N22—C23—C24	−176.7 (3)	C6—C1—N7—S8	−8.2 (4)
C23—N22—C25—C26	−56.6 (4)	C2—C1—N7—S8	172.3 (2)
C29—N22—C25—C26	−178.6 (3)	C1—N7—S8—O9	172.0 (2)
C27—N22—C25—C26	60.8 (4)	C1—N7—S8—O10	44.0 (3)
C23—N22—C27—C28	−176.8 (3)	C1—N7—S8—C11	−74.0 (3)
C25—N22—C27—C28	62.7 (4)	C3—C2—O12—C13	20.9 (4)
C29—N22—C27—C28	−55.0 (4)	C1—C2—O12—C13	−161.0 (3)
C23—N22—C29—C30	61.3 (4)	C2—O12—C13—C14	66.3 (4)
C25—N22—C29—C30	−177.1 (3)	C2—O12—C13—C18	−118.9 (3)
C27—N22—C29—C30	−57.2 (4)	C18—C13—C14—C15	−0.2 (5)
N7—C1—C2—C3	−176.0 (3)	O12—C13—C14—C15	174.3 (3)
C6—C1—C2—C3	4.3 (4)	C13—C14—C15—C16	0.1 (5)
N7—C1—C2—O12	5.9 (4)	C14—C15—C16—C17	−0.3 (6)
C6—C1—C2—O12	−173.7 (3)	C15—C16—C17—C18	0.6 (6)
O12—C2—C3—C4	176.3 (3)	C14—C13—C18—C17	0.6 (5)
C1—C2—C3—C4	−1.6 (5)	O12—C13—C18—C17	−174.1 (3)
C2—C3—C4—C5	−2.0 (4)	C16—C17—C18—C13	−0.8 (5)
C2—C3—C4—N19	178.0 (3)	C5—C4—N19—O20	176.9 (3)
C3—C4—C5—C6	2.6 (5)	C3—C4—N19—O20	−3.1 (4)
N19—C4—C5—C6	−177.4 (3)	C5—C4—N19—O21	−2.4 (4)
C4—C5—C6—C1	0.4 (5)	C3—C4—N19—O21	177.6 (3)

### Hydrogen-bond geometry (Å, º)

*Cg*1 and *Cg*2 are the centroids of the C1–C6 and C13–C18 rings, respectively.

**Table:**

*D*—H···*A*	*D*—H	H···*A*	*D*···*A*	*D*—H···*A*
C11—H11*A*···O20^i^	0.96	2.65	3.360 (4)	131
C11—H11*C*···O20^ii^	0.96	2.66	3.555 (4)	156
C17—H17*A*···N7^iii^	0.93	2.70	3.478 (4)	142
C24—H24*C*···O10	0.96	2.47	3.415 (4)	168
C25—H25*A*···O9^iv^	0.97	2.47	3.155 (5)	128
C26—H26*C*···O9^v^	0.96	2.58	3.541 (4)	175
C27—H27*B*···O9^v^	0.97	2.52	3.267 (4)	134
C14—H14*A*···*Cg*1^ii^	0.93	3.07	3.951 (5)	158
C24—H24*A*···*Cg*2^v^	0.96	2.88	3.608 (5)	134

Symmetry codes: (i) *x*+1, *y*, *z*; (ii) −*x*+1, −*y*, −*z*+1; (iii) −*x*+1, *y*−1/2, −*z*+3/2; (iv) −*x*+2, *y*+1/2, −*z*+3/2; (v) *x*, *y*+1, *z*.

## References

[bb1] Akk, G. & Steinbach, J. H. (2003). *J. Physiol.* **551**, 155–168. 10.1113/jphysiol.2003.043885 PMC2343137 12824448

[bb2] Banti, C. N., Papatriantafyllopoulou, C., Manoli, M., Tasiopoulos, A. J. & Hadjikakou, S. K. (2016). *Inorg. Chem.* **55**, 8681–8696. 10.1021/acs.inorgchem.6b01241 27513311

[bb3] Ben Brahim, K., Ben gzaiel, M., Oueslati, A. & Gargouri, M. (2018). *RSC Adv.* **8**, 40676–40686. 10.1039/c8ra07671e PMC9091414 35557925

[bb4] Bennett, A. & Villa, G. (2000). *Expert Opin. Pharmacother.* **1**, 277–286. 10.1517/14656566.1.2.277 11249549

[bb5] Dupont, L., Pirotte, B., Masereel, B., Delarge, J. & Geczy, J. (1995). *Acta Cryst.* C**51**, 507–509.

[bb6] Evans, D. J., Hills, A., Hughes, D. L. & Leigh, G. J. (1990). *Acta Cryst.* C**46**, 1818–1821.

[bb7] Farrugia, L. J. (2012). *J. Appl. Cryst.* **45**, 849–854.

[bb8] Fuentes de Arriba, L., Turiel, M. G., Simón, L., Sanz, F., Boyero, J. F., Muñiz, F. M., Morán, J. R. & Alcázar, V. (2011). *Org. Biomol. Chem.* **9**, 8321–8327. 10.1039/c1ob06126g 22057428

[bb9] Groom, C. R., Bruno, I. J., Lightfoot, M. P. & Ward, S. C. (2016). *Acta Cryst.* B**72**, 171–179. 10.1107/S2052520616003954 PMC4822653 27048719

[bb10] Ikuno, T., Chaikittisilp, W., Liu, Z., Iida, T., Yanaba, Y., Yoshikawa, T., Kohara, S., Wakihara, T. & Okubo, T. (2015). *J. Am. Chem. Soc.* **137**, 14533–14544. 10.1021/jacs.5b11046 26509741

[bb11] Kleinhaus, A. L. & Prichard, J. (1977). *J. Physiol.* **270**, 181–194. 10.1113/jphysiol.1977.sp011945 PMC1353424 915770

[bb12] Kress, H. G., Baltov, A., Basiński, A., Berghea, F., Castellsague, J., Codreanu, C., Copaciu, E., Giamberardino, M. A., Hakl, M., Hrazdira, L., Kokavec, M., Lejčko, J., Nachtnebl, L., Stančík, R., Švec, A., Tóth, T., Vlaskovska, M. V. & Woroń, J. (2016). *Curr. Med. Res. Opin.* **32**, 23–36. 10.1185/03007995.2015.1100986 26414386

[bb13] Lutz, M., Huang, Y., Moret, M.-E. & Klein Gebbink, R. J. M. (2014). *Acta Cryst.* C**70**, 470–476. 10.1107/S2053229614007955 24816016

[bb14] Oxford Diffraction (2008). *CrysAlis CCD* and *CrysAlis RED*. Oxford Diffraction Ltd, Abingdon, England.

[bb15] Rybczyńska, M. & Sikorski, A. (2023). *Sci. Rep.* **13**, 17268. 10.1038/s41598-023-44557-x PMC10570311 37828142

[bb16] Sanphui, P., Sarma, B. & Nangia, A. (2011). *J. Pharm. Sci.* **100**, 2287–2299. 10.1002/jps.22464 21491446

[bb17] Schmidt, J. E., Fu, D., Deem, M. W. & Weckhuysen, B. M. (2016). *Angew. Chem. Int. Ed.* **55**, 16044–16048. 10.1002/anie.201609053 PMC5215409 27874242

[bb18] Sheldrick, G. M. (2015*a*). *Acta Cryst.* A**71**, 3–8.

[bb19] Sheldrick, G. M. (2015*b*). *Acta Cryst.* C**71**, 3–8.

[bb20] Takekiyo, T. & Yoshimura, Y. (2006). *J. Phys. Chem. A*, **110**, 10829–10833. 10.1021/jp062911m 16970378

[bb21] Vane, J. R. & Botting, R. M. (1998). *Am. J. Med.* **104**, 2–8. 10.1016/s0002-9343(97)00203-9 9572314

[bb22] Wang, M., Ma, Y., Shi, P., Du, S., Wu, S. & Gong, J. (2021). *Cryst. Growth Des.* **21**, 287–296.

[bb23] Warnke, Z., Styczeń, E., Wyrzykowski, D., Sikorski, A., Kłak, J. & Mroziński, J. (2010). *Struct. Chem.* **21**, 285–289.

[bb24] Westrip, S. P. (2010). *J. Appl. Cryst.* **43**, 920–925.

